# A direct aircraft observation of helical rolls in the tropical cyclone boundary layer

**DOI:** 10.1038/s41598-021-97766-7

**Published:** 2021-09-21

**Authors:** Jie Tang, Jun A. Zhang, Pakwai Chan, Kaikwong Hon, Xiaotu Lei, Yuan Wang

**Affiliations:** 1 CMA/Shanghai Typhoon Institute, No. 166 Puxi Road, Shanghai, 200030 China; 2 NOAA/AOML/Hurricane Research Division and University of Miami/CIMAS, 4301 Rickenbacker Causeway, Miami, FL 33149 USA; 3 Hong Kong Observatory, Kowloon, Hong Kong China; 4 Nanjing University/School of Atmospheric Sciences, Nanjing, China

**Keywords:** Atmospheric dynamics, Fluid dynamics

## Abstract

Helical rolls are known to play a significant role in modulating both the mean and turbulence structure of the atmospheric boundary layer in tropical cyclones. However, in-situ measurements of these rolls have been limited due to safety restrictions. This study presents analyses of data collected by an aircraft operated by the Hong Kong Observatory in Typhoon Kalmaegi (1415) and Typhoon Nida (1604). Examination of the flight-level data at ~ 600 m altitude confirmed the existence of sub-kilometer-scale rolls. These rolls were mostly observed in the outer-core region. Turbulent momentum fluxes were computed using the eddy correlation method. The averaged momentum flux of flight legs with rolls was found to be ~ 2.5 times that of legs without rolls at a similar wind speed range. This result suggests that rolls could significantly modulate turbulent transfer in the tropical cyclone boundary layer. This roll effect on turbulent fluxes should be considered in the planetary boundary layer parameterization schemes of numerical models simulating and forecasting tropical cyclones.

## Introduction

Roll vortices with horizontal counter-rotating structures in the atmospheric boundary layer have axes approximately parallel to the mean environmental wind vector^1^. The directional difference between the wind vector and rolls varies from approximately − 30° to 10°. These rolls can extend throughout the whole atmospheric boundary layer and are a secondary circulation^2^. Rolls have a quasi-linear helical structure with updraft and downdraft staggered in the vertical motion. Rolls are commonly found during cold-air outbreaks and passage of sea-breeze fronts over ocean^2,3^. Clouds often form in the updraft regions and are the most-commonly used tracer of rolls, while cloud-free areas are associated with the downdraft part of the roll circulation. Characteristic length and velocity scales of roll vortices have been observed by both satellite and aircraft^2,4^. The typical vertical extent of rolls or large eddies is typically scaled by the boundary layer height, which is generally in the realm of 0.3–2 km. The wavelengths of rolls have been discussed in past literature in terms of the roll aspect ratio (i.e., wavelength divided by the boundary layer height). “Classic” roll aspect ratio in either theory or observations has a lower bound of ~ 2.4 and a maximum of 5–6 as determined by the details of the mean wind profiles modulated by the mean thermal state of the boundary layer. Previous theoretical and observational studies^5–7^ have documented the significant impact of rolls on transports of momentum, heat and moisture through the atmospheric boundary layer.

In the tropical cyclone boundary layer (TCBL), helical rolls were first observed by Wurman and Winslow^8^ (WW98 hereafter). Several studies based on radar and satellite observational studies^9–11^ investigated the spatial distribution of rolls and measured their wavelengths in the TCBL. Ellis and Businger^12^ classified roll-like structures in the TCBL into two categories: (1) streaks with a sub-kilometer scale; and (2) roll vortices with a wavelength larger than 1 km. Morrison et al.^10^ (M05 hereafter) found that rolls occurred in 35–69% of the radar volumes in four landfalling TCs. They also found that the mean wavelength of rolls is ~ 1450 m, although WW98 and Lorsolo et al.^11^ observed rolls with a much smaller wavelength that did not span the boundary layer.

Of note, Huang et al.^13^ found that km-scale and sub-km-scale rolls coexist in the TCBL by analyzing many Synthetic Aperture Radar (SAR) images. Gall et al.^14^ documented large scale (~ 10 km) rolls in landfalling hurricanes. Foster^15^ also found this type of roll signatures in SAR images taken over hurricanes. Foster provided a theoretical explanation for the formation of rolls with very different scales and why the small-scale “classic” rolls are harder to be detected than they could be in the absence of larger aspect ratio rolls. The mechanisms associated with the classic rolls may be “in effect”, but the large-scale rolls can “swamp” it. However, how rolls affect turbulent transfer remain to be understood.

In-situ observations of TCBL rolls are rare due to safety constraints and instrument limitations in high wind conditions. Zhang et al.^16^ (Z08 hereafter) for the first-time have observed rolls in the TCBL at ~ 300 m altitude through a research aircraft. They found that rolls could enhance momentum and humidity fluxes by ~ 50%. Note that Zhu et al.^14^ also observed rolls in landfalling TCs using tower observations at 10 m altitude but they did not quantify the effect of rolls on turbulent fluxes.

Besides observational studies, previous theoretical and numerical studies have investigated mechanisms for roll formation and their development in the TCBL^16–23^. Several studies have used large eddy simulation (LES) to examine the characteristics of rolls^20,23–26^. Foster^18^ (F05 hereafter) pointed out that the contribution of rolls to turbulent transport cannot be fully captured by the classical down-gradient turbulence parameterization although this classic parameterization has been widely used in numerical models. Foster’s theoretical findings were confirmed by Gao and Ginis^21,22^ who assessed effects rolls on TC intensity and structure in 3D simulations.

In this study, we present aircraft observations of rolls in the TCBL. The goal is to improve our understanding of the characteristics of rolls and their impacts on turbulent fluxes. “Dataset and method” section describes aircraft data and analysis method. “Results” section presents the observed structure of rolls and compares momentum fluxes of roll and no-roll cases. A summary of the result will be given in the last section.

## Dataset and method

### Aircraft observation and instruments

**Figure 1:**
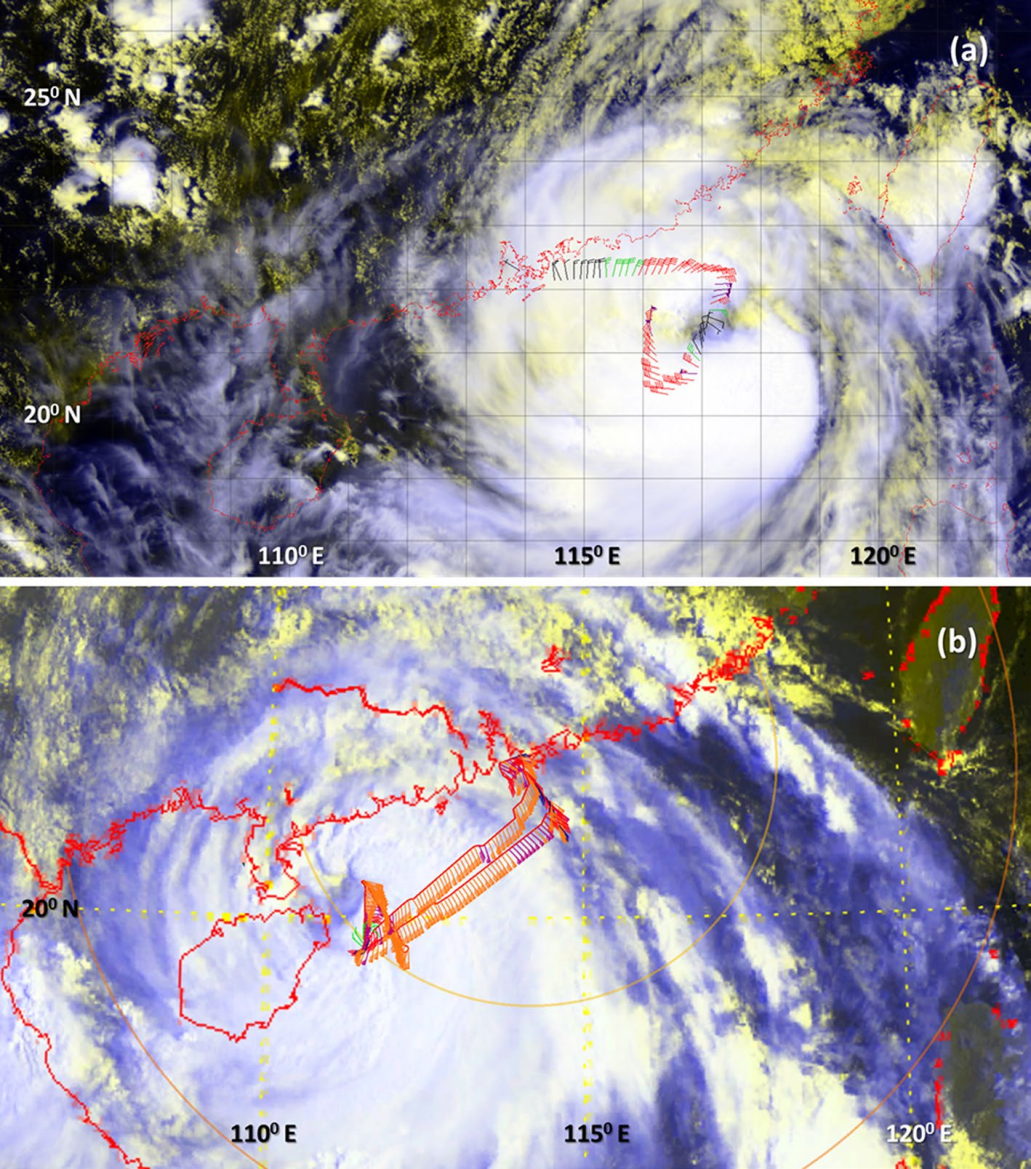
(**a**) Flight Track during Typhoon Nida and (**b**) Flight Track during Typhoon Kalmaegi. The wind bar donates the wind vector.

The Hong Kong Observation (HKO) has conducted regular observations using the Jetstream 4100 within the Hong Kong Flight Information Region (HKFIR) when typhoons posed a threat to Hong Kong in recent years^27–30^. The present study analyzes data collected by the HKO in Typhoons Kalmaegi (1415) and Nida (1604) shown in Fig. 1. The meteorological equipment on the aircraft includes a 5-hole gust probe under the wings, two Global Positioning System (GPS) antenna at the wing tips, and data processing blocks and an inertial system inside the cabin. Details of the equipment can be referred to a previous study^27^. Wind velocities, temperature, and pressure are measured with a sampling rate of 20 Hz. Table 1 shows the flight information including the time, mean aircraft altitude, maximum measured wind speed.

**Table 1:** Flight observation information of the typhoon cases.

Flight case	Start time (UTC)	End time (UTC)	Average flight height (m)	Maximum hoizontal wind (m/s)	Maximum vertical wind (m/s)	Observation frequency (HZ)
Kalmaegi	162240Sep 2014	170310Sep2014	616	77.2	10.1	20
Nida	01–1500 Aug 2016	01–1800 Aug 2016	614	51.6	12.0	20

**Figure 2:**
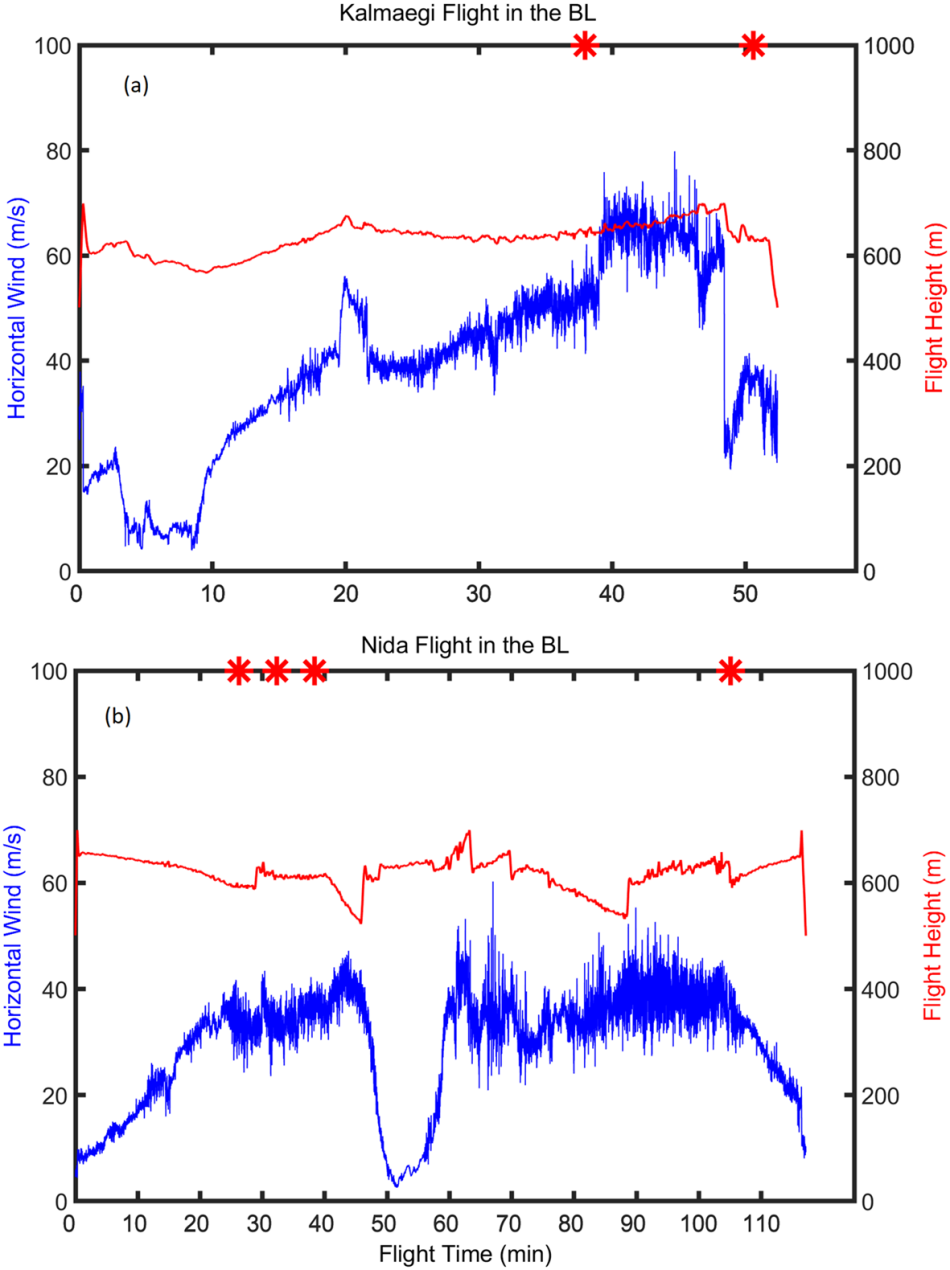
Time series of horizontal wind speed (blue) and aircraft altitude (red) in (**a**) Typhoon Kalmaegi and (**b**) Typhoon Nida.

The aircraft performed both along-wind and cross-wind legs to measure the radii of various wind strengths for TC warning purposes. As an example, Fig. 1a and b, respectively, shows the flight track into Typhoons Nida and Kalmaegi. The horizontal wind speed and aircraft altitude are shown in Fig. 2. These two flights lasted ~ 63 and 120 min in Typhoons Nida and Kalmaegi, with an averaged altitude of 616 m and 614 m (Table 1), respectively. The maximum wind speed was 77.2 and 51.6 m s^−1^, and the maximum vertical wind speed was 10.1 and 12.0 m s^−1^ in Typhoons Nida and Kalmaegi, respectively. It is noted that the mean speed of the HKO aircraft was ~ 80 m s^−1^ in Typhoon Kalmaegi and ~ 120 m s^−1^ in Typhoon Nida.

The flight into Typhoon Nida performed one set of eyewall penetration (Fig. 1). This was one of the rare cases for a research flight to penetrate the eyewall of a severe typhoon within the boundary layer over the Pacific Ocean. Aircraft observations in Typhoons before the Nida mission were only conducted in the TC ambient region, including those conducted as part of the DOSTAR experiment^31^. Although the flight into Typhoon Kalmaegi could not reach the eyewall region due to safety constraints, a stepped ascent flight pattern was conducted during this flight in the boundary layer of the outer core region.

### Data analysis method

In this study, we split the flight into legs every 2–3 min for flux calculations. We identify along- and cross-wind legs using an angle between the flight heading and the mean wind direction parameter (${\upalpha }$) as follows:

$${\upalpha } = \frac{d}{r}\left\{ {\begin{array}{*{20}c} { \ge 0.5} & {Cross\;wind\;leg} \\ { < 0.5} & {Along\;wind\;leg} \\ \end{array} } \right.$$

Here $r$ is the length of a leg and $d$ donates the radial distance at the beginning and end of the leg. If ${\upalpha }$ is larger than 0.5, we define this flight leg as a cross-wind leg and vice versa. This threshold value was defined to restrain the directional difference between the across flight direction and mean wind direction to be no less 30 degrees. We identified a total of 54 cross-wind legs and 31 along-wind legs during the two typhoon flights.

Previous roll studies^9,14^ pointed out that the roll axis was usually aligned with the mean wind vector. In addition, the most distinguish characteristics of rolls is the positive–negative staggered horizontal vorticity and vertical velocity. This implied that cross-wind legs have a higher chance to detect rolls. The eyewall penetration legs are usually cross-wind legs, however, it is very difficult to conducted this type of observations within the TCBL again due to safety constraints. During the CBLAST experiment, although a total of 10 cross-wind legs were attempted, only 2–3 legs showed a roll-like structure. Only one leg of aircraft data clearly confirmed roll structure in the BL of Hurricane Isidore (2002) as documented by Z08. Of note, Z08 used both spectral and wavelet analysis methods to extract the information of rolls from flight-level data. Z10 also used the wavelet analysis method to detect rolls in the surface layer of landfalling hurricanes by using tower data. Here we use the same methods as used in these previous studies^12,16^ to analyze the flight-level wind data in typhoons and investigate characteristics of rolls based on the wavelet package by Torrence and Compo ^32^. Momentum fluxes are calculated using the eddy correlation method.

## Results

**Figure 3:**
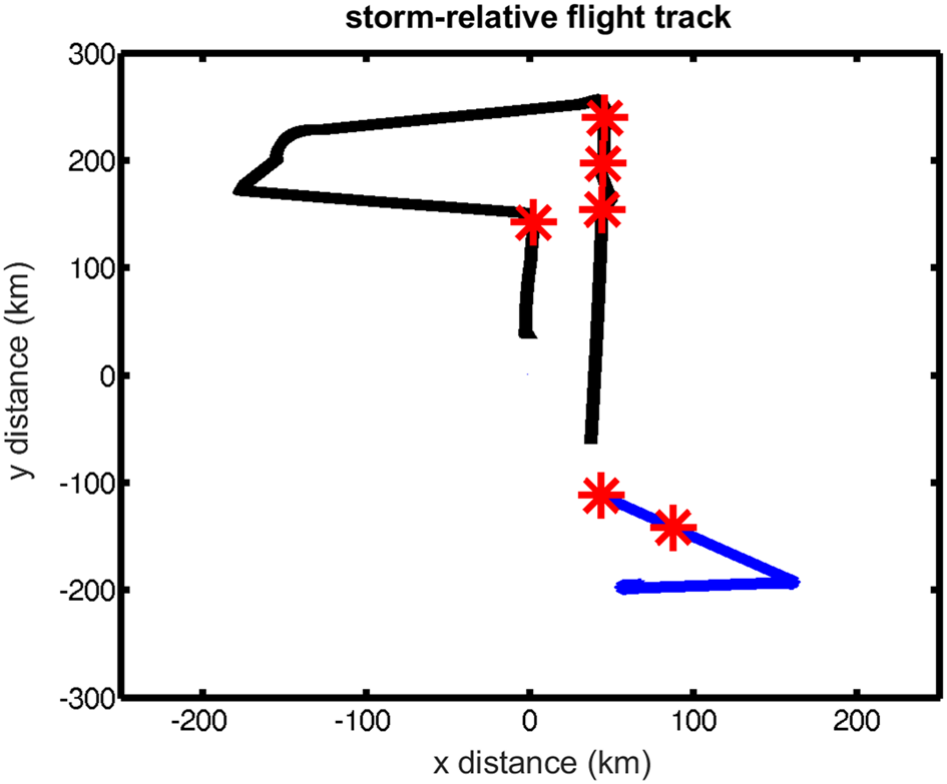
Legs of roll cases (red stars) during the flights in Nida (black) and Kalmaegi (blue). The flight tracks are plotted in a storm-relative framework with (0, 0) representing the storm center.

The locations of the six roll legs are shown in Fig. 3 along with the flight tracks in the two storms. All these roll legs are cross-wind legs that are located in the region within a radius range of 150 km to 250 km. This result may imply that rolls in TCBL are more easily detected in the outer core region than close to the RMW in a similar manner as in Z08. No roll case was found in the eye or eyewall region in our study.

**Figure 4:**
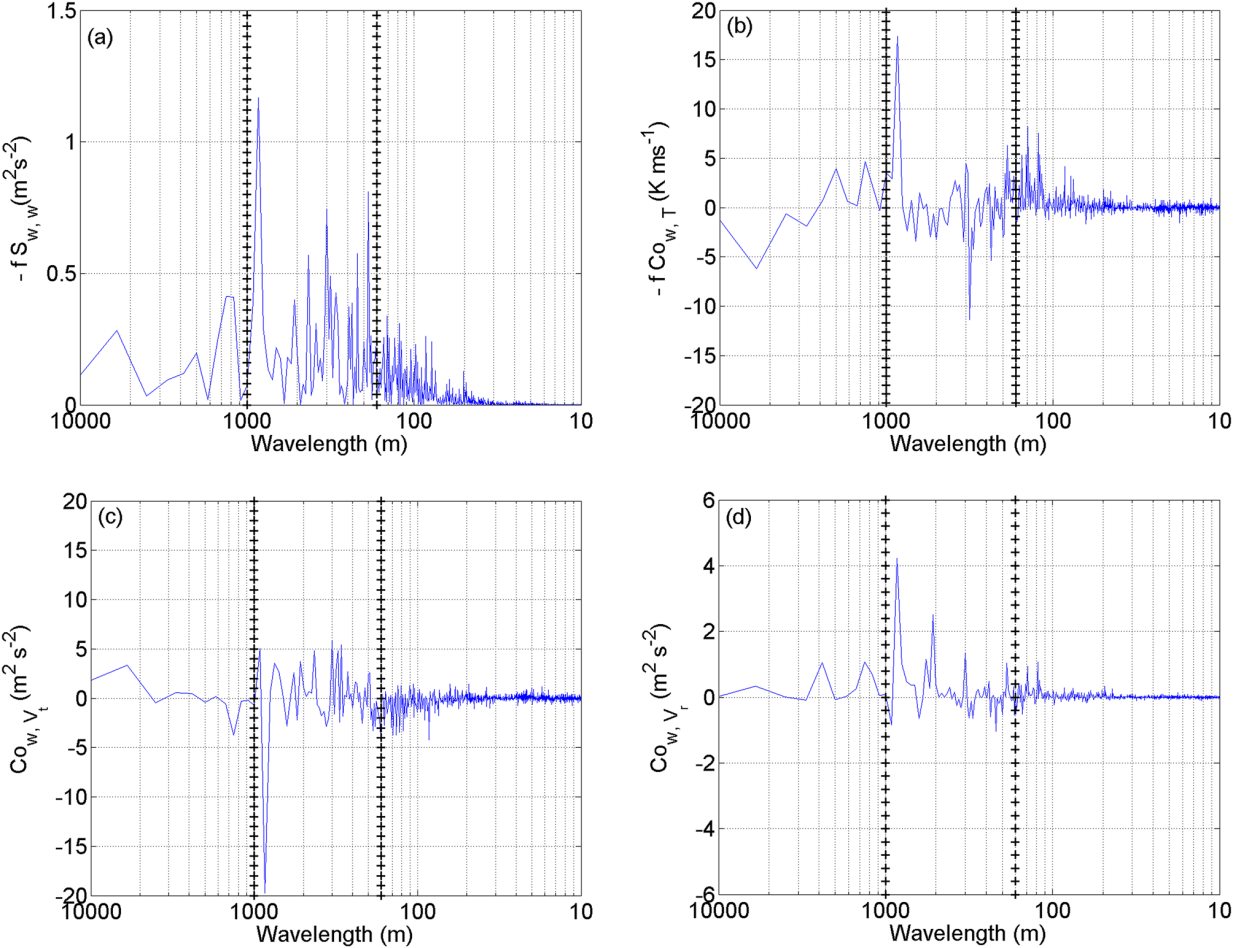
Plots of (**a**) spectra of vertical velocity (w), (**b**) cospectrum of w and temperature (T), (**c**) cospectrum of w and tangential wind speed (V_t_), and (**d**) cospectrum of w and radial wind speed (V_r_) as a function of wavelength.

The spectrum of vertical velocity (*w*), cospectrum of *w* and temperature (*T*), cospectrum of *w* and tangential wind speed (*V*_*t*_), and cospectrum of *w* and radial wind speed (*V*_*r*_) of a typical roll leg are shown in Fig. 4a–d, respectively. There are several significant peaks in the *w* spectrum. The strongest signal is at a wavelength of 960 m in all spectral and cospectral plots, indicating the dominant wavelength of rolls in this case assuming this radial leg is perfectly toward the storm center. The other spectral peak at the wavelength of 480 m which is half of the roll wavelength is also substantial in these spectral and cospectral plots. This result indicates that sub-kilometer scale rolls with different wavelengths could co-exist in the TCBL. This result also supports the theoretical finding of Foster^18^ who showed that the lowest-order nonlinear contribution in the model is the first harmonic which contributes at lower energy than the primary wave. These rolls detected here are similar to the wind streaks nearly aligned with the mean wind direction seen in radar observations^7,9,11^. Rolls are generated due to the long-term quasi-equilibrium shear instability, and hence they are larger and “fill” the TCBL^15^.

**Figure 5:**
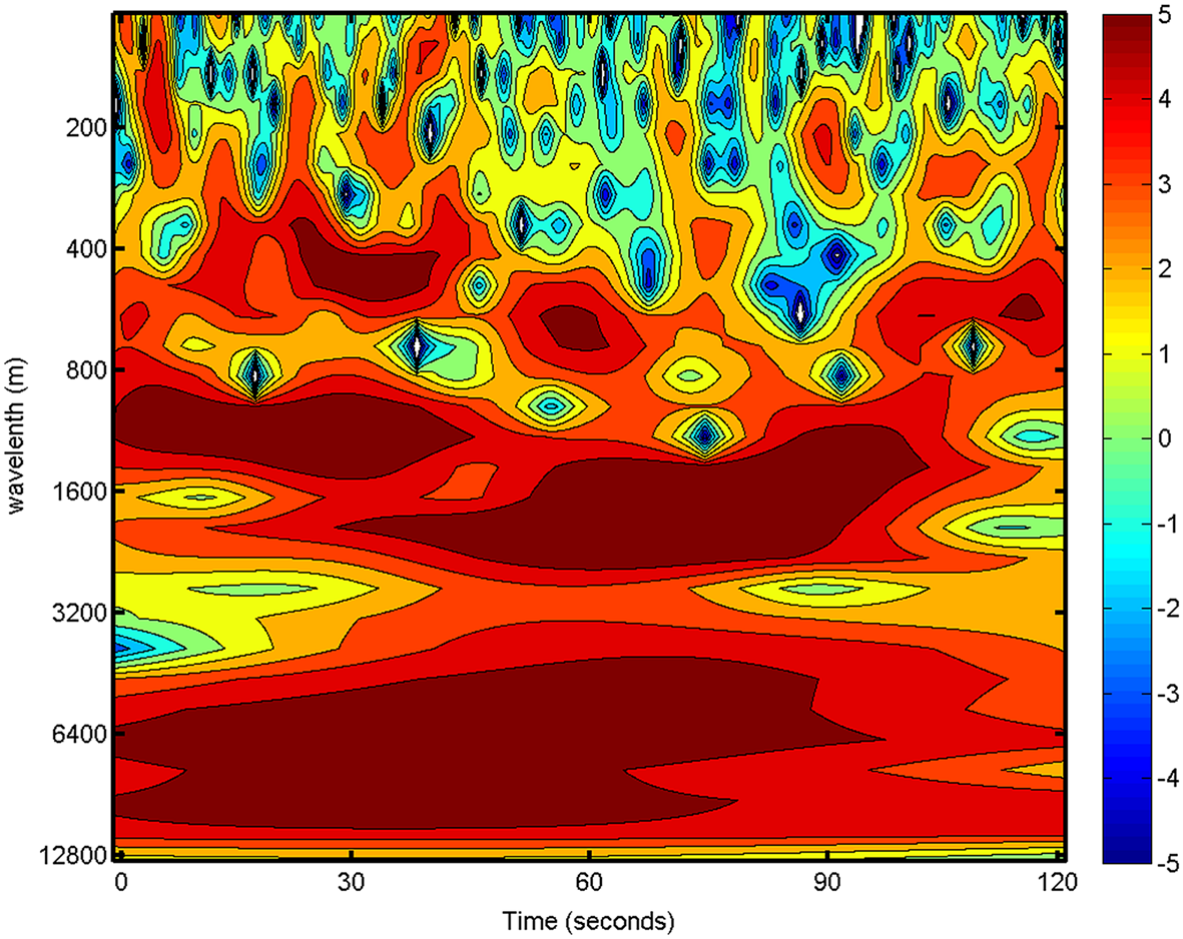
Wavelet coefficient of the vertical velocity for the roll leg as in Fig. 4 during the flight in Typhoon Nida.

The roll-like structure can also be observed by the enhanced energy patterns in the wavelet coefficient of *w* in Fig. 5 for the same cross-wind leg as in Fig. 4. Two wavelet energy peaks are noticed in the wavelength range of 800–3000 m, indicating relative large-scale coherent structures. There are also periodic peaks in the wavelength range of 300–800 m indicating sub-kilometer scale rolls. This result is similar to the wavelet analysis of Z08^11^. Such coherent features are only observed in the cross-wind legs in our study again suggesting the rolls are generally aligned with mean wind direction^1,9,13^.

**Figure 6:**
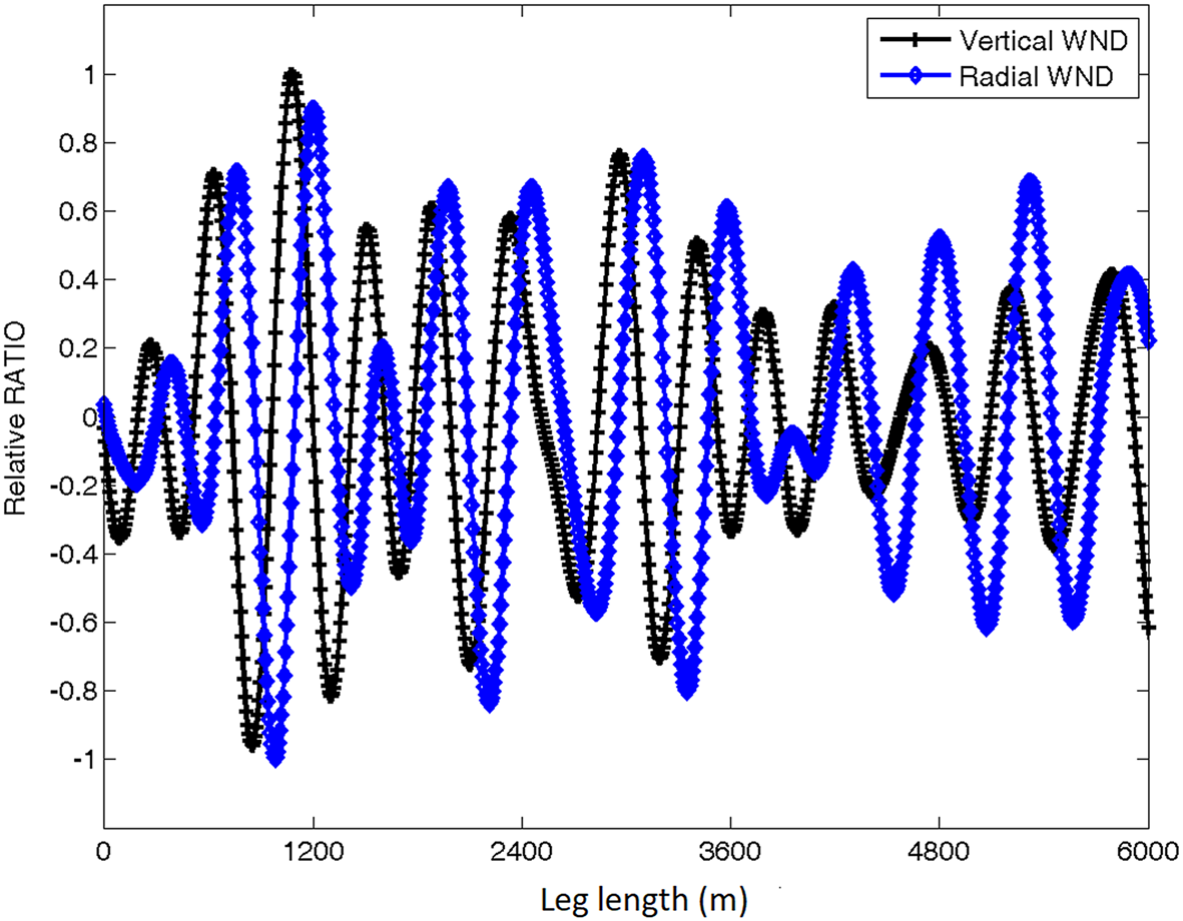
Plots of the vertical velocity normalized by the peak updraft (black) and radial wind velocity normalized by the maximum inflow strength (blue) for the roll leg in Typhoon Nida.

The vertical velocity normalized by the peak updraft and radial wind velocity normalized by the maximum inflow strength are shown in Fig. 6 for the same radial leg as in Fig. 4. Note that the radial component was also scaled by the maximum radial velocity perturbation from the leg mean. The normalized wind fields show a positive–negative staggered distribution. Furthermore, the staggered distribution of vertical and radial velocities shows an exact phase difference of π/2. The wavelength of the staggered structure is in the range of 400–1600 m which is consistent with the roll wavelengths seen in the spectral and wavelet analyses.

**Figure 7:**
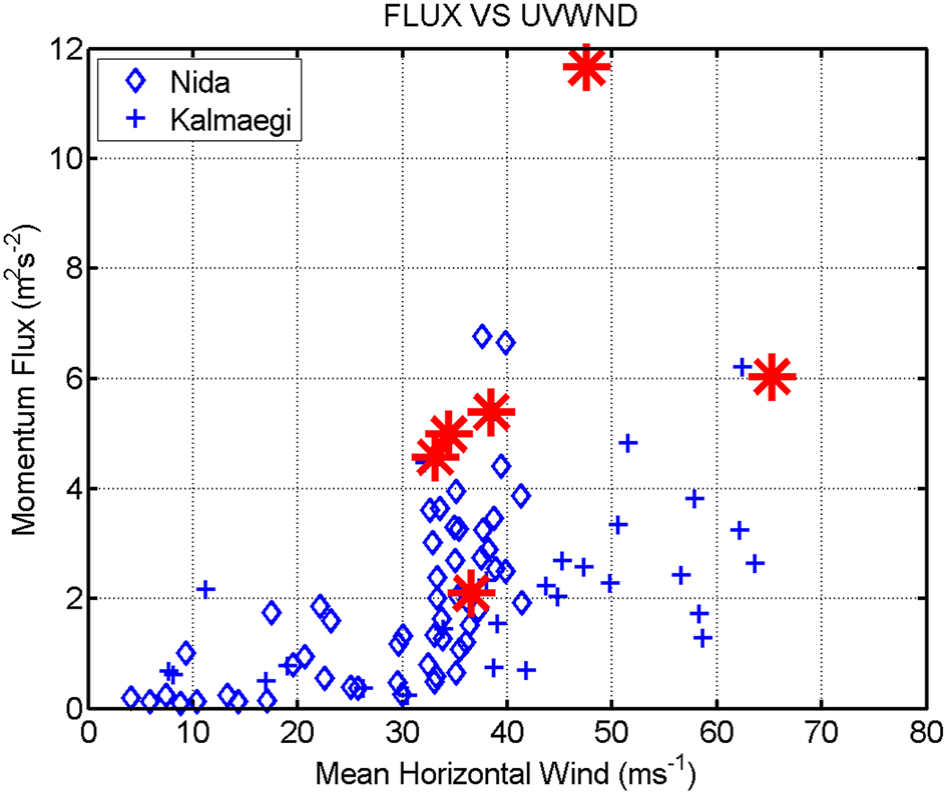
Plot of the momentum fluxes from all legs in Typhoons Nida (diamond) and Kalmaegi (+) as a function of the mean horizontal wind speed of each leg. Red stars represent the roll legs.

Our result generally supports the prevalence conclusion partly drawn by M05 who found ~ 35%to 69% fraction of observation cases may contain rolls by Doppler radars. To evaluate the potential role of the TCBL rolls in turbulent transport, we compare the momentum fluxes of the rolls to those of legs without rolls (Fig. 7). Our result shows that the mean momentum flux of the roll legs is significantly larger than that of legs without rolls. The ratio of the mean momentum flux of the roll vs. non-roll legs is 2.27. This ratio is larger than that found by Z08 who only detected one roll leg but quite agree with the simulation study based on large eddy simulation (LES) by Glendening^23^ in general atmosphere boundary layer and by Zhu^11^ in TC. However, our estimate relative roll effect on momentum flux is close to that of M05 although the magnitudes of the fluxes are much smaller than those of M05 using Doppler radar data.

## Conclusion and discussion

TCBL rolls have been mainly observed by Doppler radar and SAR images in the past, while the in-situ observations have been scarce. It is difficult to detect rolls using ground-based fixed observations such as towers due to the movement of the positive–negative staggered structure of boundary rolls^1,7^. There was only one case study of rolls directly observed by aircraft in the TCBL by Z08. Mechanisms of roll formation and characteristics of rolls thus remain to be explored^7,9,12–16^.

**Figure 8:**
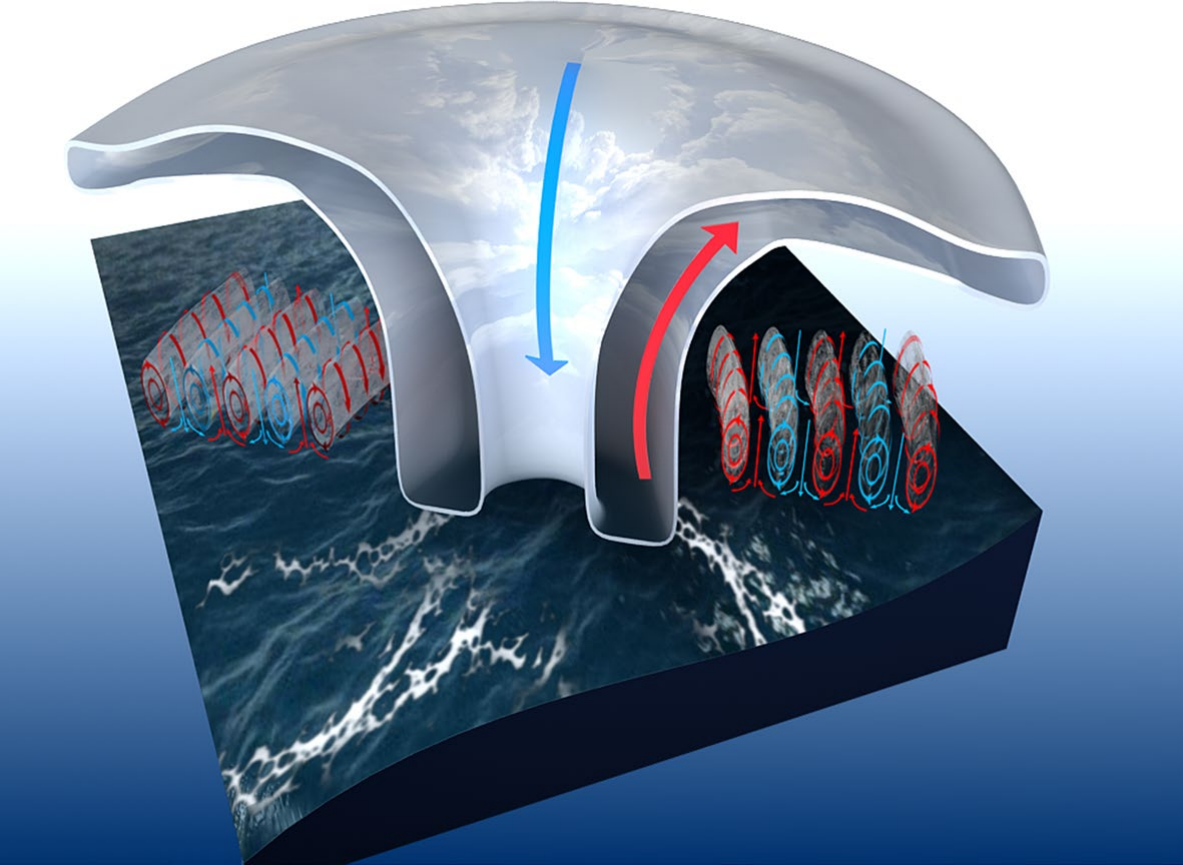
Schematic diagram of roll distribution in the tropical cyclone boundary layer based on aircraft observations in Typhoons Nida and Kalmaegi.

Since 2012, the HKO has conducted aircraft observations in typhoons. In this study, we analyzed flight-level fast-response wind data during two successful flights into Typhoon Kalmaegi (1415) and Typhoon Nida (1604). A total of six flight legs with roll structure were detected. All these roll cases were found in the outer core region (R = 150–250 km) from the cross-wind legs. These rolls have a wavelength of sub-kilometer to kilometer scales. Figure 8 shows a conceptual diagram for the roll distribution in the two storms we tudied here. Our analyses suggest that radial legs (i.e., cross-wind legs) are more efficient for roll detection than along-wind legs in the TCBL given that rolls tend to align with the mean wind direction.

Our result shows that the momentum fluxes of legs with rolls on average are ~ 2–3 times those of legs without rolls, confirming the important effect of rolls on turbulent transport in the TCBL. This roll-effect on turbulent mixing is recommended to be included in the TCBL parameterization schemes in numerical models which has been indicated by previous LES study^20–23^. Given that rolls are only detected in the cross-wind legs, certain along-wind legs that may encounter rolls could not detect them. This may explain why several along-wind legs ‘without’ rolls still have relatively high values of momentum fluxes (4–7 m^2^/s^2^, c.f. Figure 7). Note that a parallel flight that is 1/4 or 1/2 of the roll wavelength away might find a different flux over the same distance at the same relative part of the storm. If the leg was at a small angle to the rolls, the flux modulation would be aliased into wavelengths beyond the reasonable length for flux calculation. In a practical sense, aircraft measurements of roll flux contributions can only be made in cross-roll legs.

Previous SAR observations of BL rolls showed that the roll signal was alternating bright and dark backscatter regions^13,15,16^. To the lowest order, these parallel bright and dark bands in the SAR images are associated with locally higher and lower surface wind stress. Thus, a roll parallel leg would be preferentially sampling fluxes that are lower or higher than an areal mean that encompass many instances of rolls. Future observational studies of TCBL rolls using cross-wind flight patterns are recommended to quantify flux difference between roll versus no-roll cases.

To fully understand the effects of rolls on momentum, heat and moisture fluxes, more in-situ observations in the TCBL with high-quality turbulence sensors are required in the future. Given the safety constraints and severe environment for crewed aircraft observations, a combination of crewed and uncrewed aircraft observations would be ideal for roll detection and flux observations^33–35^. Since the roll feature could potentially cause large damages near the surface, improved understanding of their distribution and impacts in TCs is crucial for advancement of operational forecasts^36–38^.

## Data Availability

The datasets during and/or analysed during the current study available from the corresponding author on reasonable request.
